# Supplementary material for: Neuron-specific chromosomal megadomain organization is adaptive to recent retrotransposon expansions
Source: Nat Commun. 2021 Dec 13;12:7243. doi: 10.1038/s41467-021-26862-z (PMC8669064; doi:10.1038/s41467-021-26862-z)
Supplement: Supplementary file 1 — Supplementary Information [file 41467_2021_26862_MOESM1_ESM.pdf]

Supplementary Materials for

NEURON-SPECIFIC CHROMOSOMAL MEGADOMAIN ORGANIZATION IS ADAPTIVE TO RECENT RETROTRANSPOSON EXPANSIONS

Sandhya Chandrasekaran, Sergio Espeso-Gil, Yong-Hwee Eddie Loh, Behnam Javidfar, Bibi Kassim, Yuhao Dong, Yueyan Zhu, Lucy K. Bicks, Haixin Li, Prashanth Rajarajan, Cyril J. Peter, Esperanza Agullo-Pascual, Marina Iskhakova, Molly Estill, Bluma J. Lesch, Li Shen, Yan Jiang*, Schahram Akbarian*

Correspondence to: schahram.akbarian@mssm.edu

This PDF file includes:

<u>*Figures S1 to S21*</u>

Figure S1 *NeuN+ Hi-C clusters*
Figure S2 *NeuN- Hi-C clusters*
Figure S3 *Correspondence of NeuN+ subcompartment loci across replicates*
Figure S4 *Subcompartment connectivity across independent studies reveals consistent patterning in adult forebrain neurons.*
Figure S5 *Trans interactions by subcompartment*
Figure S6 *Trans interactions among B2 chromosomal megadomains*
Figure S7 *Hi-C trans profiles in NeuN+ and NeuN-*
Figure S8 *Trans interactions occur less frequently among B_2^{NeuN+} loci in NeuN- as compared to NeuN+*
Figure S9 *Gene clusters in B_2^{NeuN+}*
Figure S10 *CTCF binding motif enrichment*
Figure S11 Strain- and sex-specific heatmap of Hi-C subcompartment interactions
Figure S12 *PacBio SMRT long-read sequencing of IAPEzi de novo integration sites in adult mouse cortex and round spermatids*
Figure S13 *IAP gag DNA FISH in NeuN+ and NeuN- nuclei*
Figure S14 *Hi-C circos plots from NeuN+ of Camk-Cre^{+}, Setdb1$^{2lox/2lox}$ mutant mice (adult cortex)*
Figure S15 *Histone methylation at Setdb1-sensitive genes.*
Figure S16 *GOs for elevated transcripts in Camk-Cre^{+}, Setdb1$^{2lox/2lox}$ mutant vs. control adult cortex*
Figure S17 *Microglia RNA-Seq and ATAC-Seq*
Figure S18 *NeuN+ and NeuN- H3K9me3 (KO/WT) for ERV classes (I, II, and III)*
Figure S19 *NeuN+ and NeuN- H3K9me3 (KO/WT) for non-ERV repeat categories*
Figure S20 *Mouse/human subcompartment comparisons*
Figure S21 *Inter-chromosomal contacts in human neurons are enriched with (H)ERV retroelements*

<u>*Tables S1 to S5*</u>

Table S1 *ChIP-Seq metrics*
Table S2 *PacBio qPCR primers*
Table S3 *PacBio oligonucleotide capture probes*
Table S4 *RNA-Seq metrics*
Table S5 *Discordant + chimeric RNA-Seq*

Figure S1

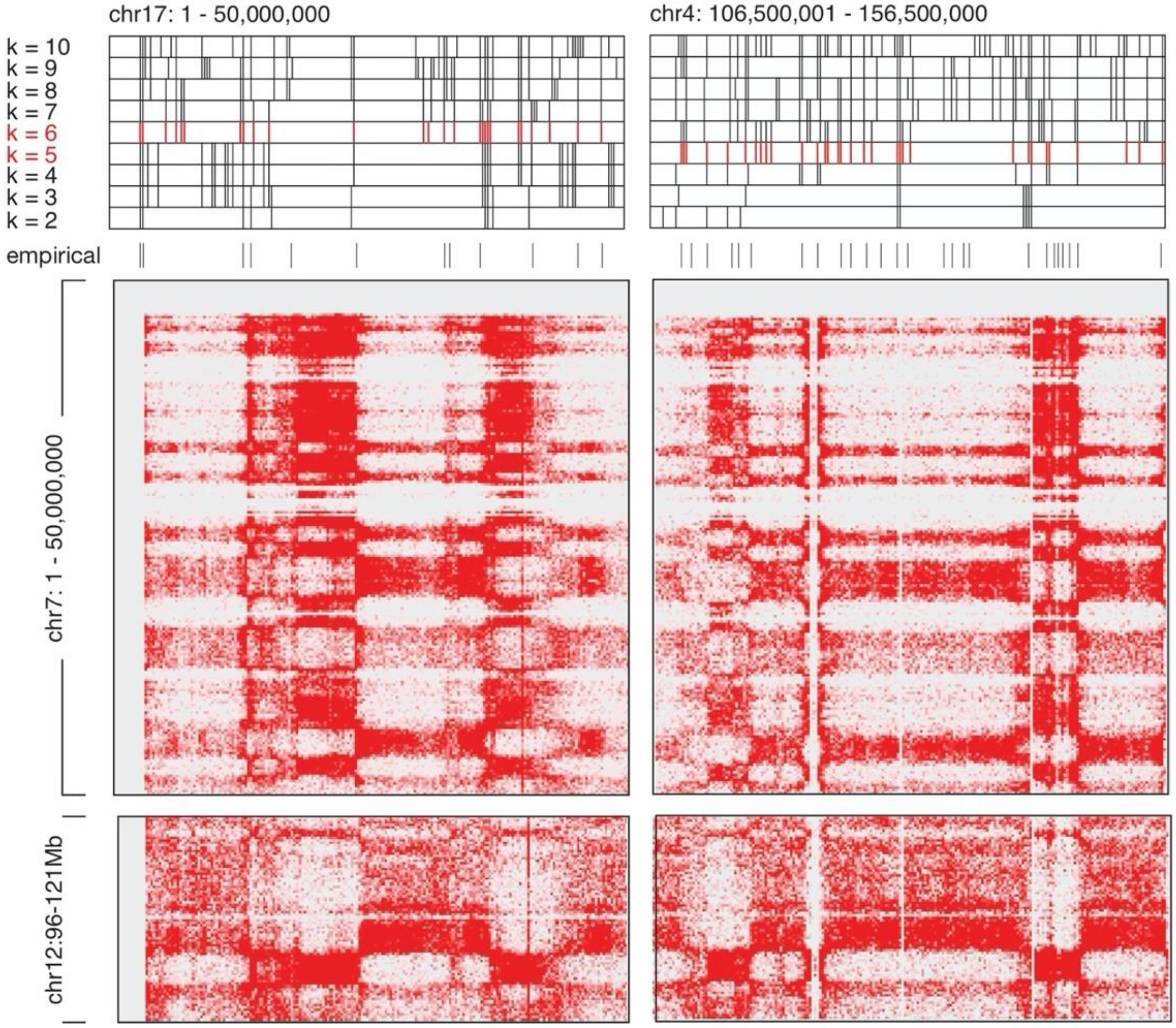

Figure S1. NeuN+ Hi-C clusters. Observed/expected HiC interaction matrices (n=4, 2F/2M) for NeuN+ chromatin were used to extract values using *hicdump*. K-means clustering was performed separately for odd and even chromosomes (see *Methods*). Multiple cluster numbers were tested, ranging from k=2 (bottom) to k=10 (top). Visual corroboration of cluster boundaries was performed against empirically determined boundaries (gray) based on the visual inspection of the HiC *trans* contact maps, as described in the k-means clustering strategy of Rao, et. al. (2014)[11]. Odd chromosomes (see chr17) were most concordant with k=6 (red), while even chromosomes (see chr4) were most concordant with k=5 (red). To match and collapse these clusters across all chromosomes, any cluster comprising <2% of the loci within the genomic space were first removed, and the remaining clusters (n=4 within odds, 4 within evens) were combined in order of size.

Figure S2

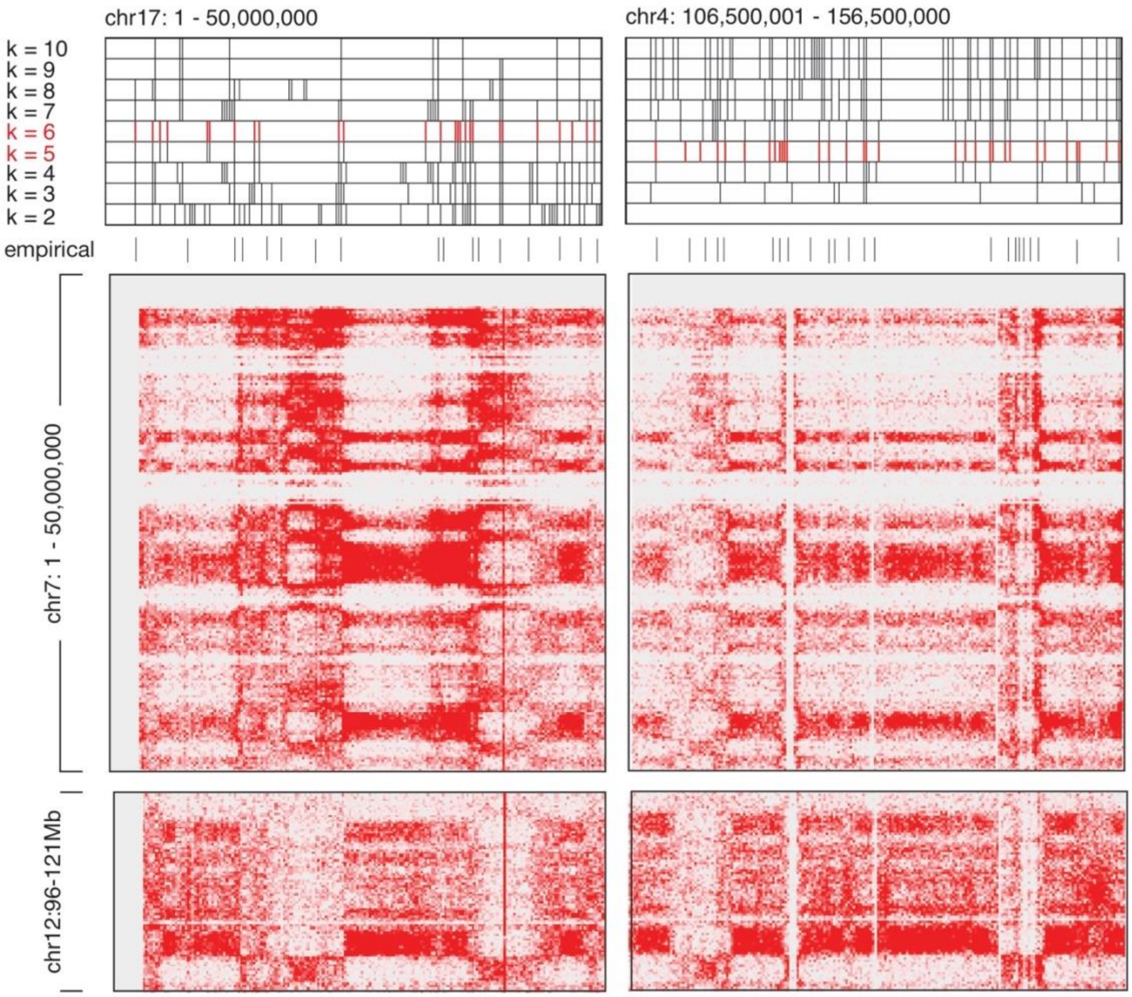

Figure S2. NeuN- Hi-C clusters. Observed/expected HiC interaction matrices (n=4, 2F/2M) for NeuN- chromatin were used to extract values using *hicdump*. K-means clustering was performed separately for odd and even chromosomes (see *Methods*). Multiple cluster numbers were tested, ranging from k=2 (bottom) to k=10 (top). Visual corroboration of cluster boundaries was performed against empirically determined boundaries (gray) based on the visual inspection of the HiC trans contact maps, as described in the k-means clustering strategy of Rao, et. al. (2014) [11]. Odd chromosomes (see chr17) were most concordant with k=6 (red), while even chromosomes (see chr4) were most concordant with k=5 (red). To match and collapse these clusters across all chromosomes, any cluster comprising <2% of the loci within the genomic space were first removed, and the remaining clusters (n=4 within odds, 4 within evens) were combined in order of size.

Figure S3

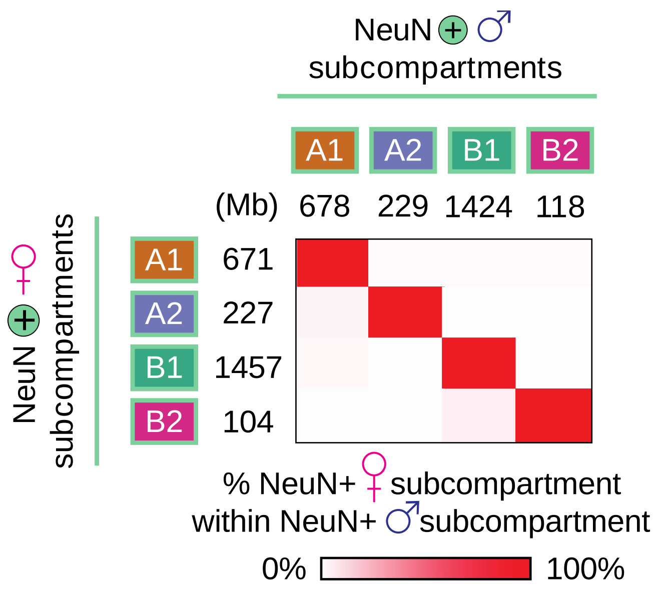

Figure S3. Correspondence of NeuN+ subcompartment loci across replicates. Percent overlap of shared genomic coordinates within each subcompartment combination between the female (n=2, rows) and male (n=2, columns) Hi-C files. Color scale indicates percent of 250kb bins within the female NeuN+ subcompartment shared within the denoted male NeuN+ subcompartment; 0% (white), 100% (red). NeuN+ subcompartments were independently called in female (n=2) and male (n=2) Hi-C files; the respective sizes of these subcompartments are denoted in Mb along the axes.

Figure S4

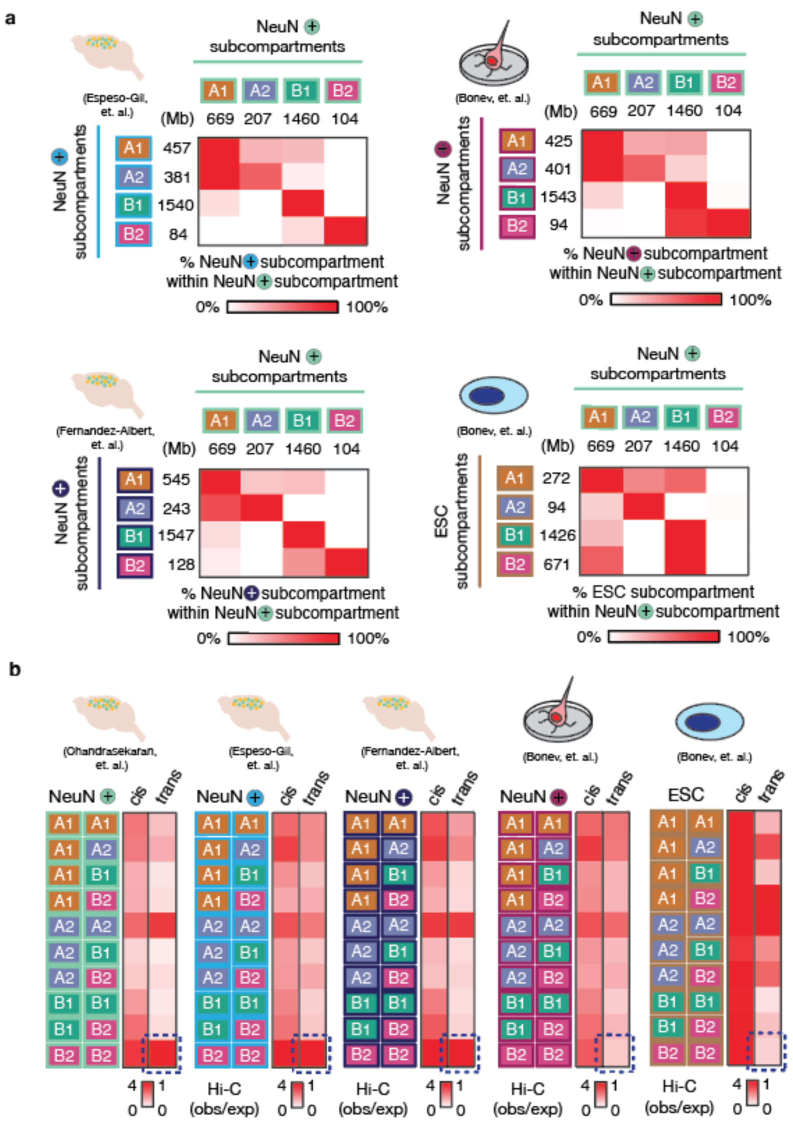

Figure S4. Subcompartment connectivity across independent studies reveals consistent patterning in adult forebrain neurons. (A) Neuronal subcompartments from independently published datasets largely correspond to the NeuN^{+} subcompartments of the current study (shown as reference set, horizontal axis). Independent datasets: (*Top left)* Neurons from adult mouse cortex (n=2) [Espeso-Gil, et. al. (2021)[12]]; (*bottom left*) Neurons from adult mouse hippocampus (n=2) [Fernandez-Albert, et. al. (2019)[13]]; (*top right*) Immature neurons from neuronal culture (n=2) [Bonev, et. al. (2017)[14]]; (*bottom right)* ESCs (n=4) from culture [Bonev, et. al. (2017)[14]]. Genomic extents of each subcompartment as indicated. Percent overlap of coordinates from each subcompartment with each of the NeuN^{+} reference subcompartments are represented on the indicated color scale. (B) Heatmap of mean Hi-C (observed/expected) between loci comprising the designated subcompartments in *cis* and *trans*; 250kb resolution. NeuN^{+} Hi-C of present study (*Chandrasekaran, et al.*) is shown on the far left. Notice highly similar connectivity pattern, including B2-B2 contacts, among the three Hi-C datasets from adult mouse brain neurons (highlighted with the dashed boxes, blue).

Figure S5

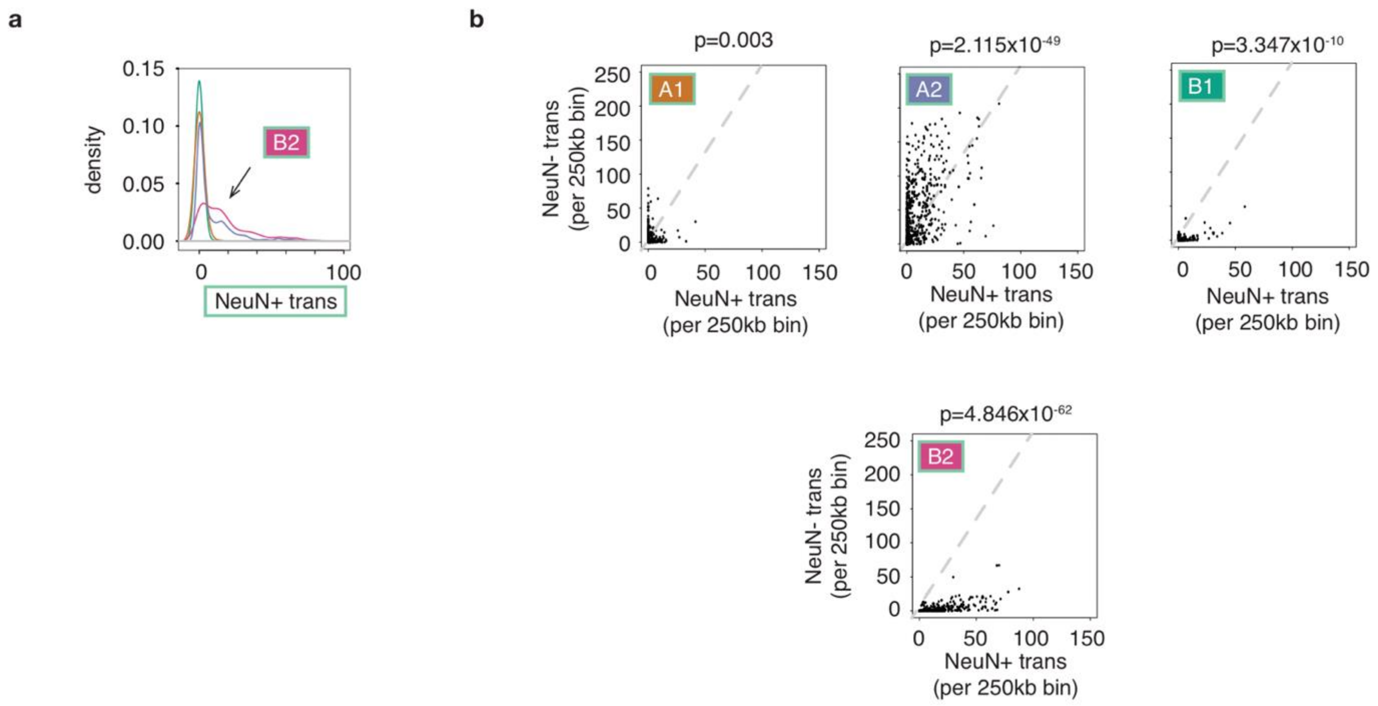

Figure S5. Trans interactions by subcompartment. (A) Density plot representing the NeuN+ *trans* interactions occurring in 250kb bins comprising each of the four subcompartments. Colors: A1 (orange), A2 (purple), B1 (green), B2 (pink). Note the dichotomy in *trans* interactions across subcompartments, with A1 and B1 plots narrowly peaking near 0, and A2 and B2 plots (highlighted with arrow), are flatter and more skewed towards higher *trans* interactions (x-axis). (B) Scatterplots of mean NeuN+ *trans* (x-axis) vs. NeuN- *trans* (y-axis) interactions (n=4/group) for loci comprising the NeuN+ subcompartments. Dotted line in gray is the genome-wide expected line calculated from the mean trans- interactions called in NeuN+ and NeuN-. P-values were calculated using paired Student's t-testing (two-sided) between the observed and expected NeuN- *trans* interactions per 250kb bin genome-wide based on the observed NeuN+ *trans* interactions at that bin.

Figure S6

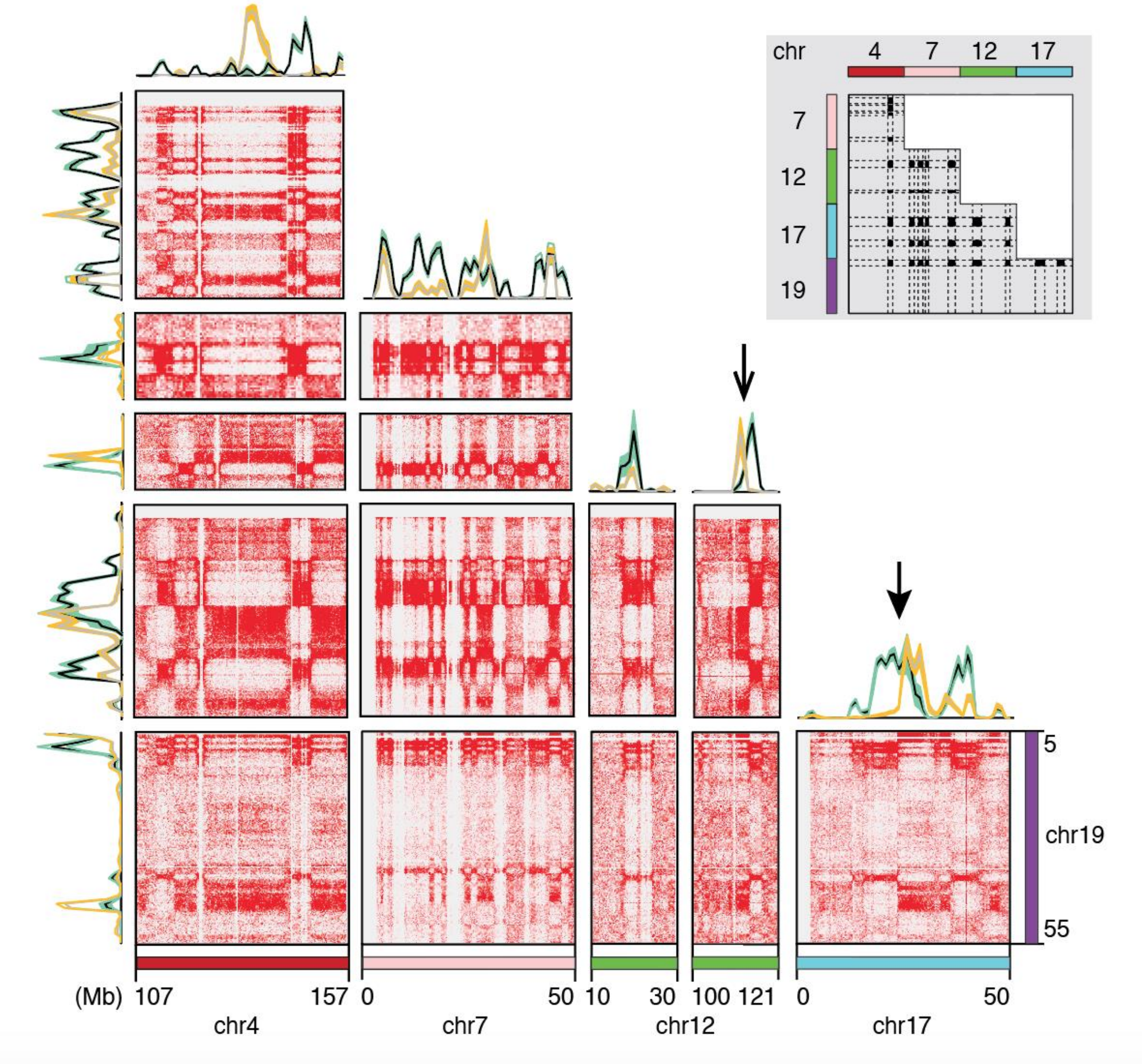

Figure S6. ***Trans*** **interactions among B2 chromosomal megadomains.** Select Hi-C pairwise matrices highlighting chromosomal megadomains of B2 subcompartment loci engaging in neuron-specific interactions in trans. Traces on the axes of these matrices indicate the number of significant HOMER interactions (threshold $p < 1x10^{-50}$) each locus along the chromosome participates in genome-wide; green line traces represent NeuN+, and orange line traces represent NeuN-. The schematic at the top right of figure highlights the portions of each chromosome that have been magnified and incorporated into the figure. Note sharp boundaries of Hi-C densities (red) coincident with boundaries of genome segments participating in *trans* interactions only in NeuN+ vs. in both NeuN+ and NeuN- (thin arrow on chr12; thick arrow on chr17).

Figure S7

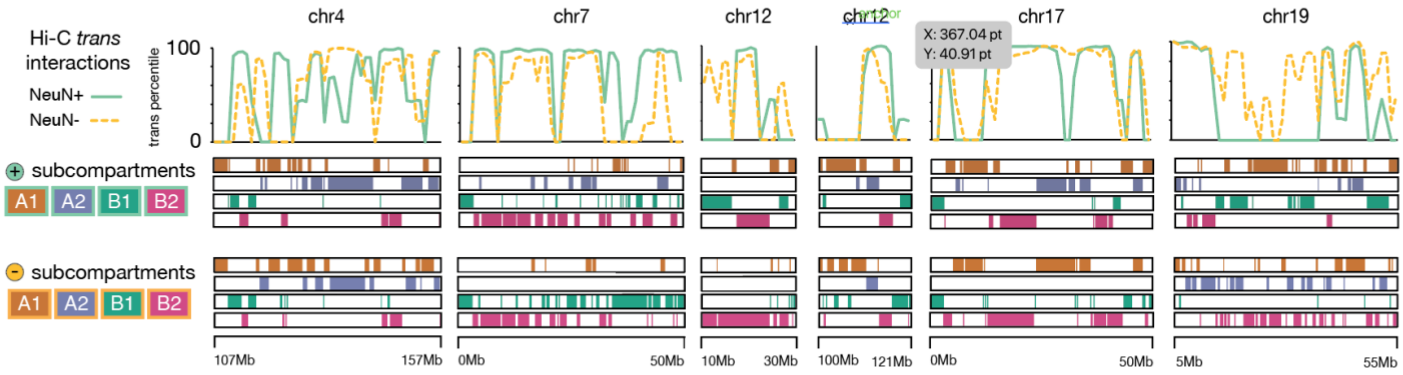

Figure S7. Hi-C *trans* profiles in NeuN+ and NeuN-. Hi-C pairwise *trans* contact maps for NeuN+ (green) and NeuN- (orange) across several chromosomes. X-axis denotes genomic coordinates in 1Mb increments; y-axis denotes the mean percentile (n=4/group) of significant HOMER (threshold: $p < 10^{-50}$) *trans* interactions called within each 1Mb bin along the represented chromosomal segment, by respective cell type. Subcompartment designations in NeuN+ (top) and NeuN- (bottom) included. Note this plot is identical to Figure 1H but has been converted into percentiles to superimpose data from both cell types onto the same axis.

Figure S8

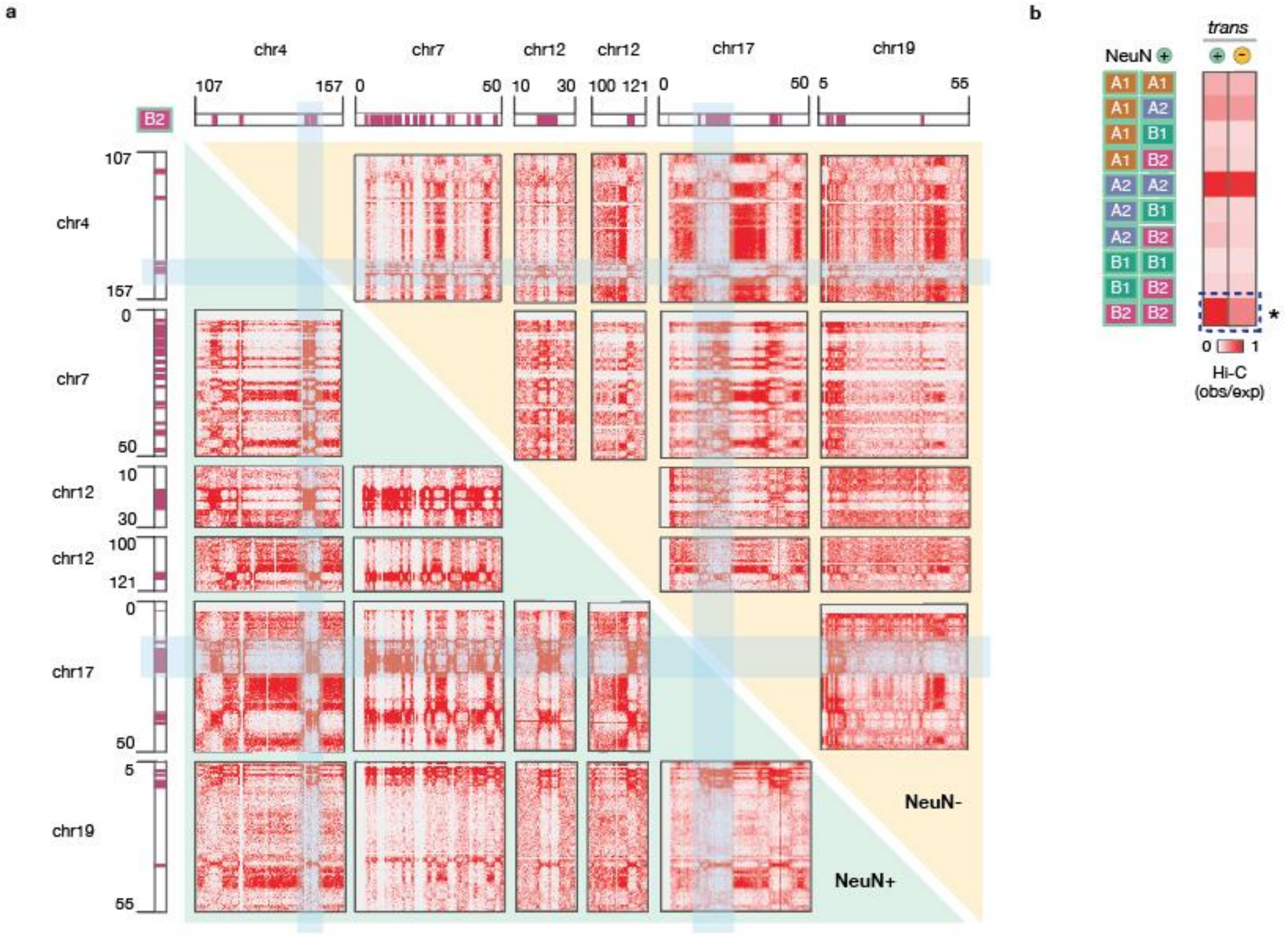

Figure S8. ***Trans* interactions occur less frequently among $B2^{NeuN+}$ loci in NeuN- as compared to NeuN+.** (A) Select Hi-C pairwise matrices highlighting interaction frequencies among chromosomal megadomains comprising $B2^{NeuN+}$ subcompartment loci engaging in neuron-specific interactions in *trans*. $B2^{NeuN+}$ chromosomal megadomains displayed as tracks (highlighted in pink) along the axes of the overall matrix. Matrices highlighted in green denote NeuN+ Hi-C interaction maps; matrices highlighted in orange denote NeuN- Hi-C interaction maps. Select interactions between $B2^{NeuN+}$ loci are highlighted in blue to denote contrasting interaction frequencies at the same coordinates in NeuN+ vs. NeuN-. (B) Heatmap of mean Hi-C (observed/expected) between loci comprising the designated NeuN+ subcompartments in *trans* (left) and *cis* (right) in NeuN+ (n=4) and NeuN- (n=4); 250kb resolution. Notice the reduced *trans* mean in NeuN- vs. NeuN+ (highlight with dashed box, blue) for interactions occurring between $B2^{NeuN+}$ loci; statistical significance determined using Student's t-test ($p < 10^{-300}$, two-sided, paired).

Figure S9

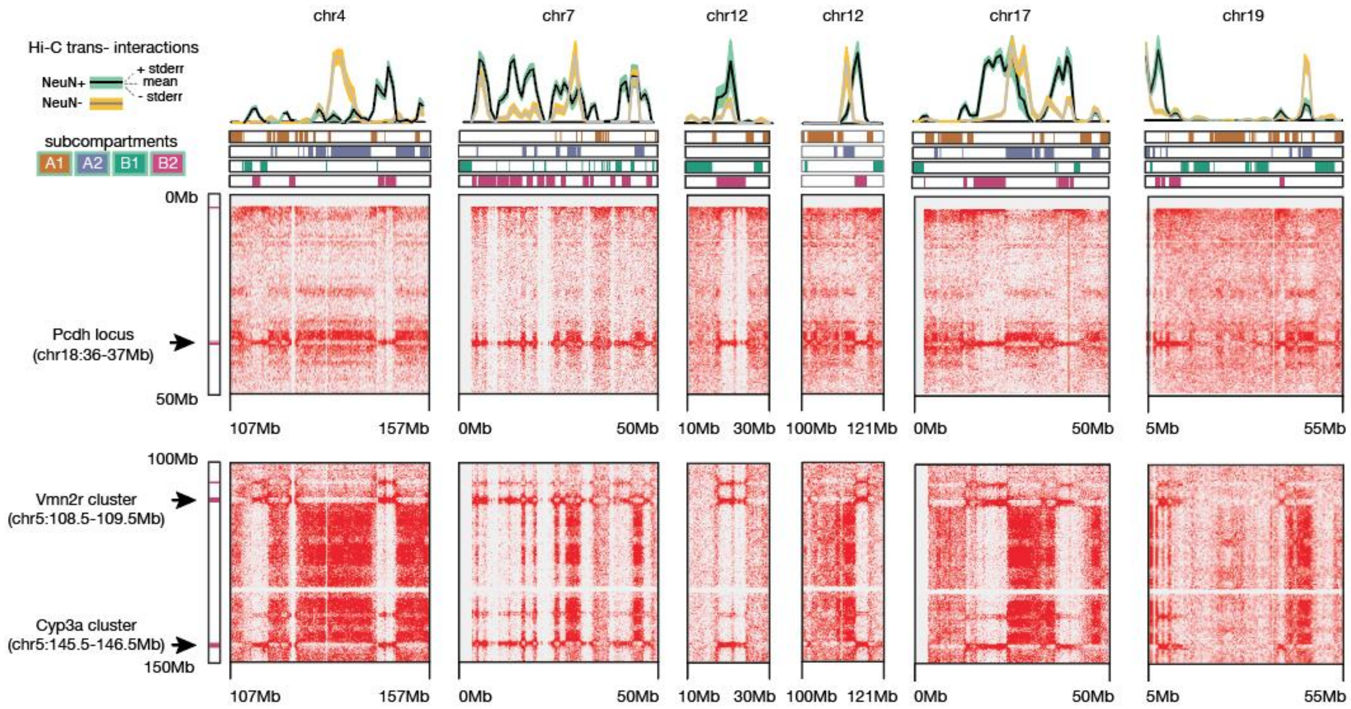

Figure S9. Gene clusters in B2^{NeuN+}. Hi-C pairwise contact maps encompassing multiple gene clusters (y-axis). Note the marked coincidence of observed Hi-C interactions corresponding to each gene cluster with the B2 subcompartment loci (pink).

Figure S10

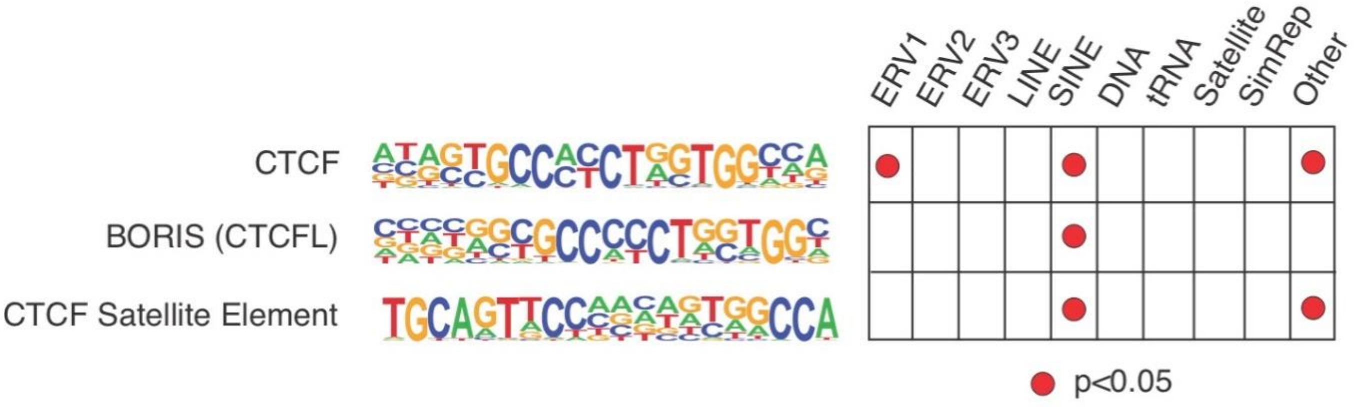

Figure S10. CTCF binding motif enrichment. Binding motif enrichment for CTCF and CTCF-related architectural proteins (BORIS and CTCF Satellite Element) across repetitive element categories using HOMER binomial testing (one-sided). Note that ERV2s do not collectively enrich for CTCF and CTCF-related binding proteins, in contrast to SINE elements which are significantly associated (as corroborated in the literature); -log(p-values) as depicted.

Figure S11

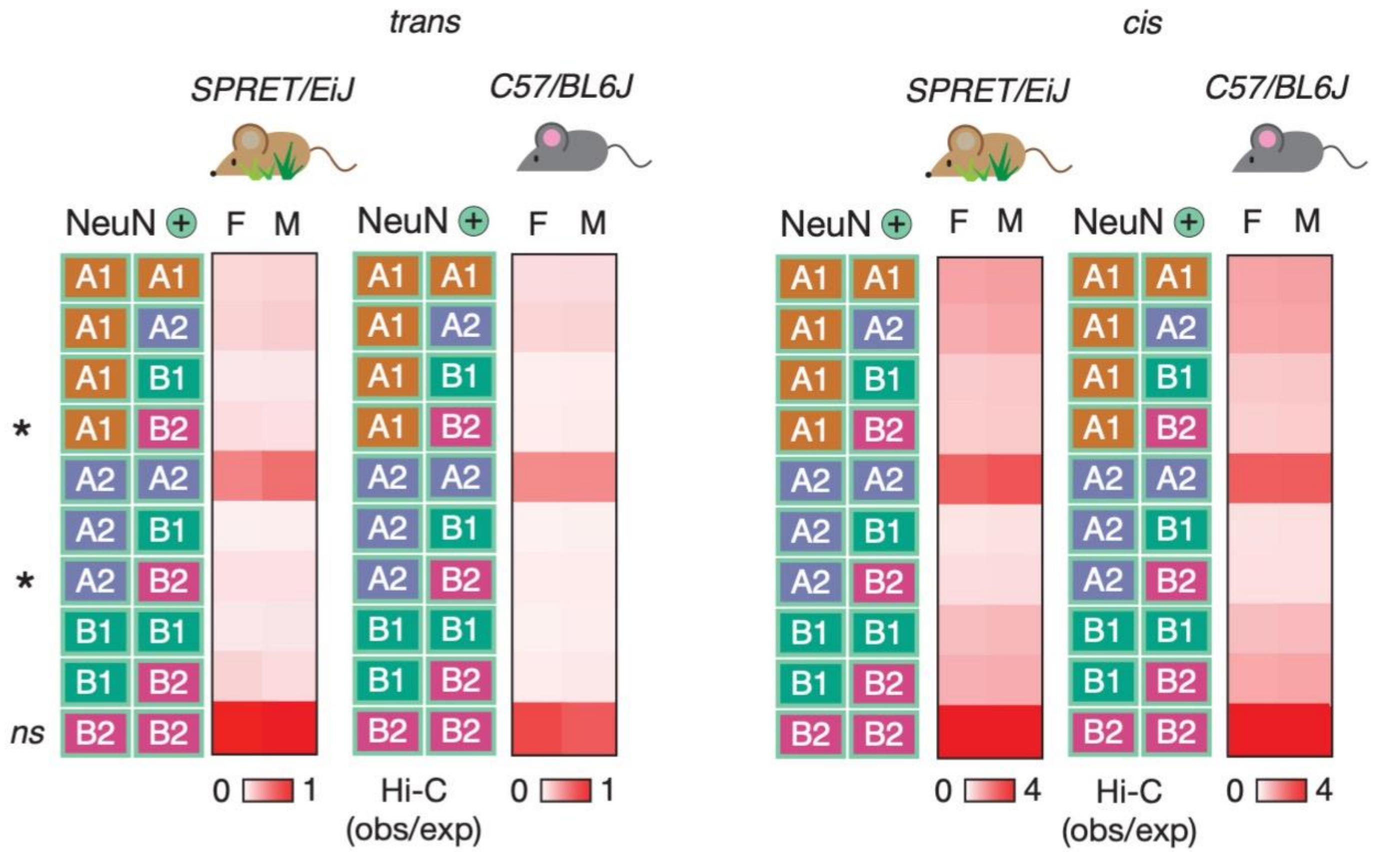

Figure S11. Strain- and sex-specific heatmap of Hi-C subcompartment interactions. Heatmap for mean Hi-C (observed/expected) between loci comprising the designated NeuN+ subcompartments (SPRET/EiJ or C57/BL6J) in *trans (left)* and *cis (right)*; 250kb resolution. Asterisks (*) indicate subcompartment interactions that differed significantly between SPRET/EiJ and C57/Bl6J ((*) $p < 0.05$; Student's t-testing, paired by sex, two-sided); ns indicates not significant. (A1:B2 p= 0.03379389; A2:B2 p= 0.04796373). Notice the overall similar *cis* and *trans* interaction profiles among subcompartments in both strains; however, B2 loci demonstrate significantly increased *trans* frequencies of interactions with A1 and A2 subcompartments in SPRET/EiJ vs. C57/Bl6J.

Figure S12

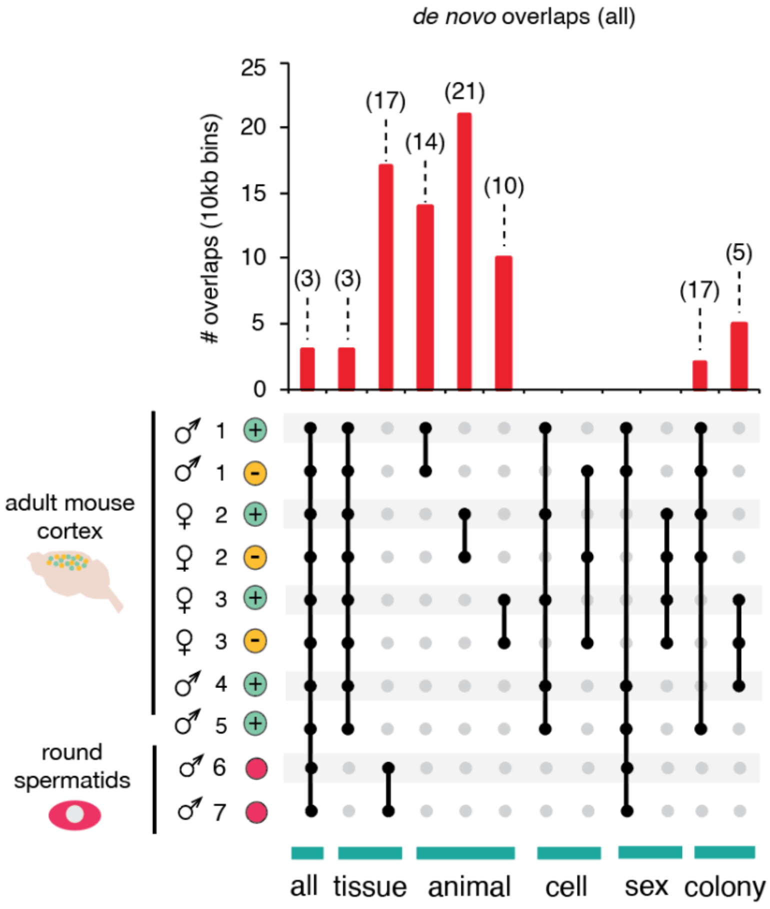

Figure S12. PacBio SMRT long-read sequencing of IAPEzi *de novo* integration sites in adult mouse cortex and round spermatids. Number of *de novo* overlaps, including full-length (10kb resolution), shared among the different subsets of biological specimens tested (depicted as red bars). Samples are grouped by tissue, animal, cell type, sex, and colony, as indicated with the black connected dots.

Figure S13

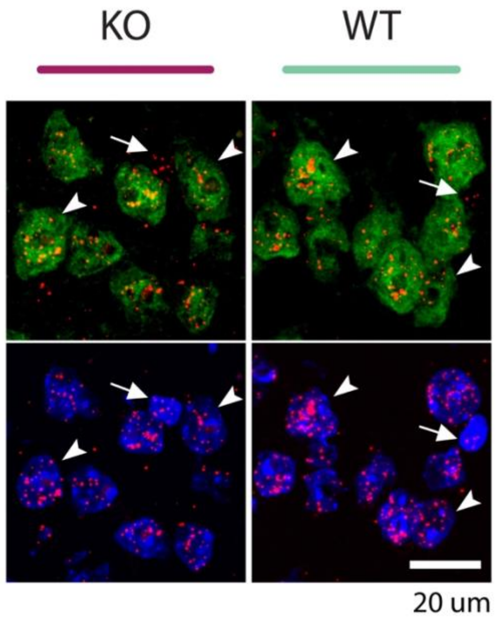

Figure S13. IAP gag DNA FISH in NeuN+ and NeuN- nuclei. IAP gag DNA FISH signal in coronal sections in *Setdb1* KO vs. WT adult cortex. Counterstained with NeuN (green) and DAPI (blue) as indicated. Arrows and arrowheads mark representative examples of NeuN+ and NeuN- nuclei, respectively.

Figure S14

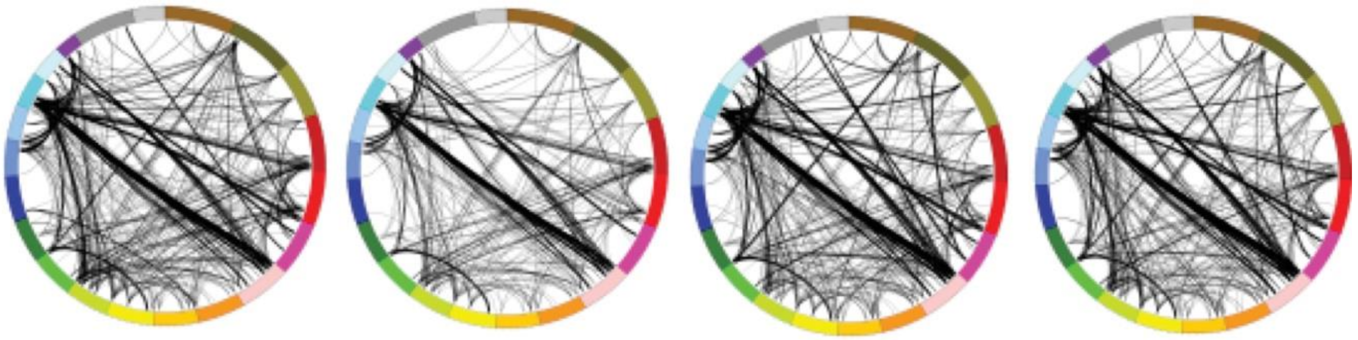

Figure S14. Hi-C circos plots from NeuN+ of *Camk-Cre*$^{+}$*, Setdb1*$^{2lox/2lox}$ mutant mice (adult cortex). Circos plots depicting Setdb1-cKO *trans* chromosomal interactions determined by HOMER v4.8. Autosomes (chr1-19) and chrX/Y are depicted along the periphery of the circle in clockwise fashion, with chr 1 (brown, 12:00), Significance threshold: $p < 1x10^{-50}$.

Figure S15

	mouse NeuN + > - (H3K9me3)		KO < WT (H3K9me3)	
	↑DEGs	↓DEGs	↑DEGs	↓DEGs
A1	4	1	1	0
A2	3	0	1	0
B1	4	3	0	0
B2	36	0	2	0

Figure S15. Histone methylation at Setdb1-sensitive genes. DEGs between Setdb1-cKO and WT are minimally enriched with NeuN+ specific H3K9me3 at baseline and are minimally altered in H3K9me3 enrichments upon Setdb1 loss. Total upregulated DEGs (764); total downregulated DEGs (324). (Left) Each value in the table denotes the number of genes per category (upregulated or downregulated DEG) with significant H3K9me3 in NeuN+ > NeuN- in WT (diffReps, $p < 0.001$) by subcompartment. (Right) Each value in the table denotes the number of genes per category (upregulated or downregulated DEG) with significant H3K9me3 changes in KO NeuN+ as compared to WT NeuN- (diffReps, $p < 0.001$) by subcompartment.

Figure S16

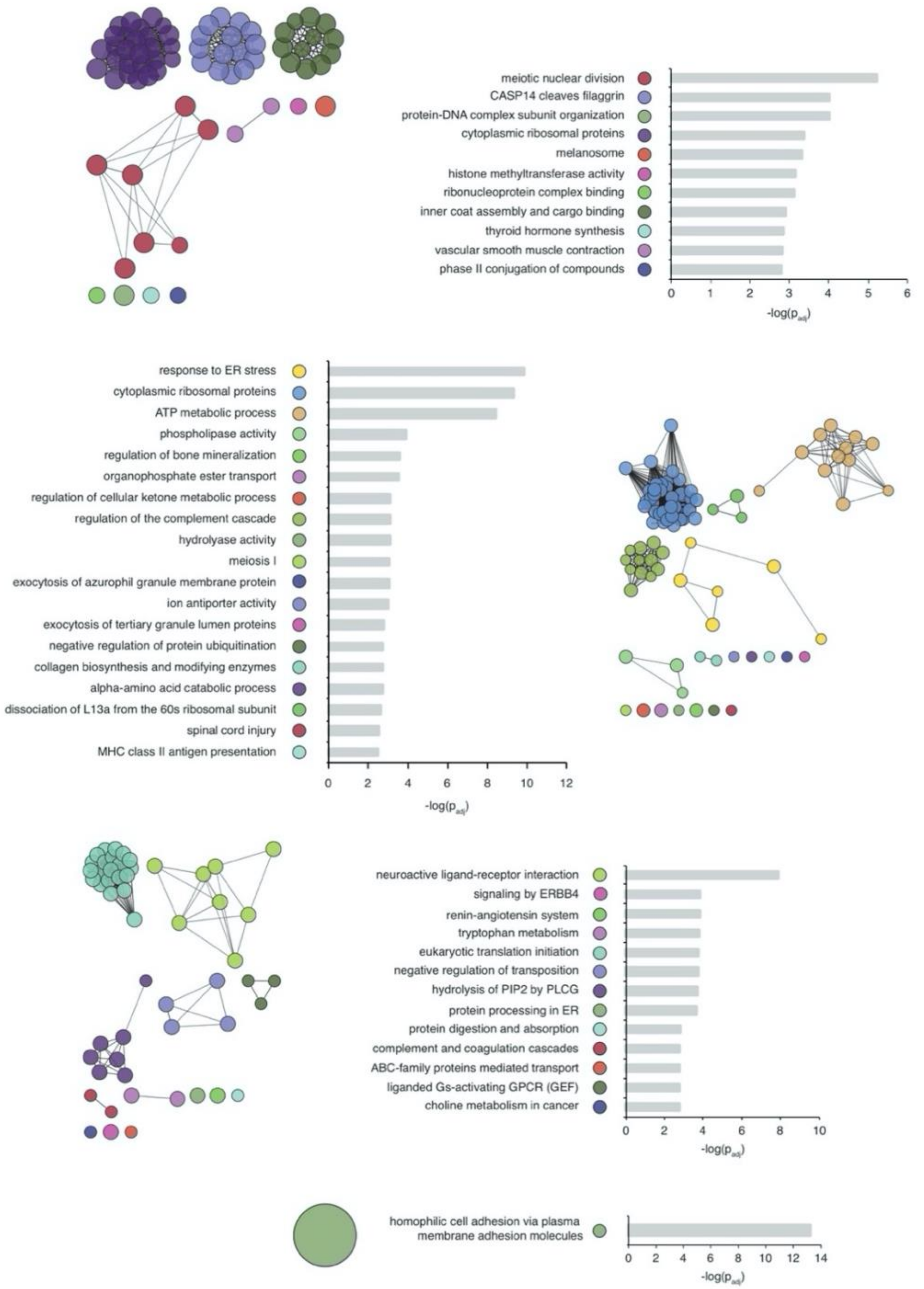

Figure S16. GOs for elevated transcripts in *Camk-Cre*$^{+}$, *Setdb1*$^{2lox/2lox}$ mutant vs. control adult cortex. (n=6/group) Top ten categories displayed per subcompartment, with corresponding enrichment significance for each GO category (-log(p_{adj})) (ClueGO (v2.5.1) for Cytoscape (v3.6.1, right-sided hypergeometric test analysis with Benjamini-Hochberg p-value correction)). From top to bottom, modules and GO analyses correspond to A1, A2, B1, and B2. Gene modules are depicted with connected nodes.

Figure S17

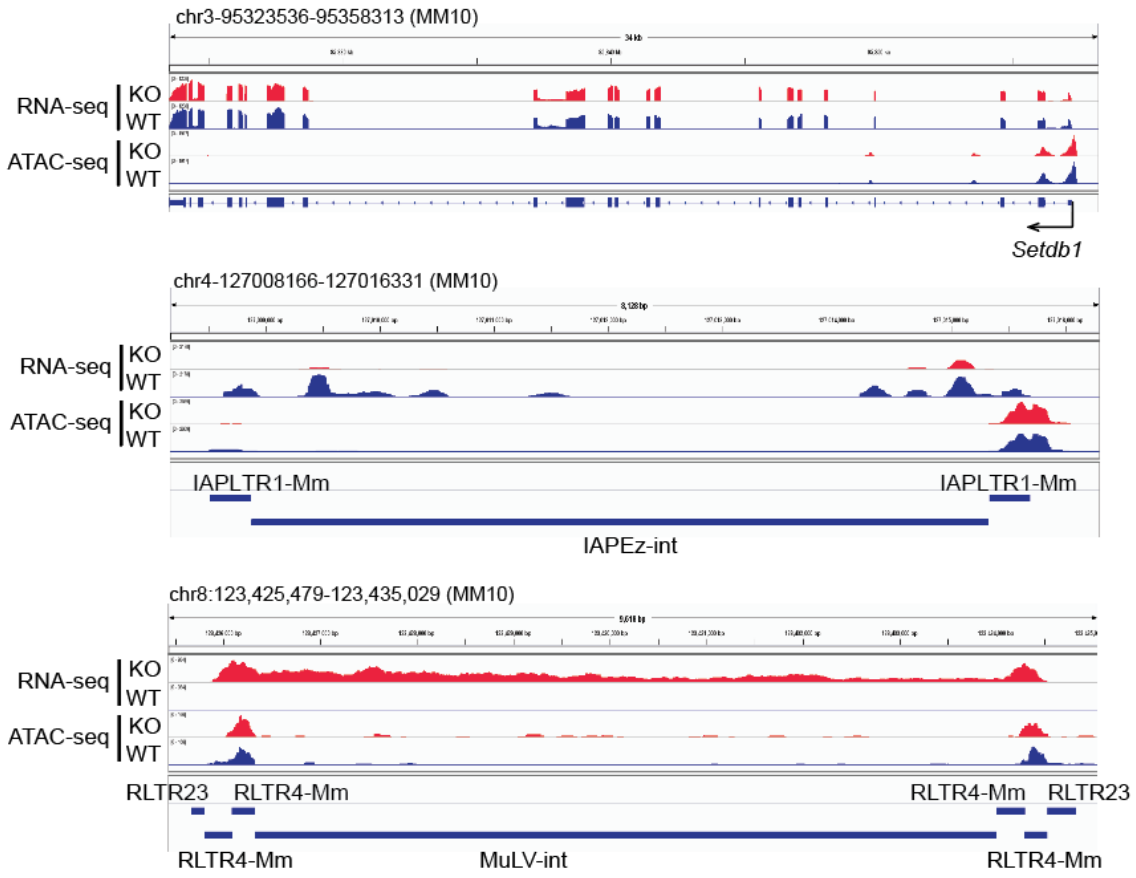

Figure S17. Microglia RNA-Seq and ATAC-Seq. Representative images show signal at (Top) *Setdb1* gene, (Middle) IAPEz-int, and (Bottom) ERV1-MuLV loci from microglia-specific RNA-seq and ATAC-seq in *Setdb1-CK-cKO* and controls. Notice no change for *Setdb1* transcription, especially exon III that was ablated in our neuronal knockout system. Similarly, note no change in the chromatin accessibility at the locus. Notice the decrease of IAPEz-int and increase of MuLV transcripts at these two specific loci.

Figure S18

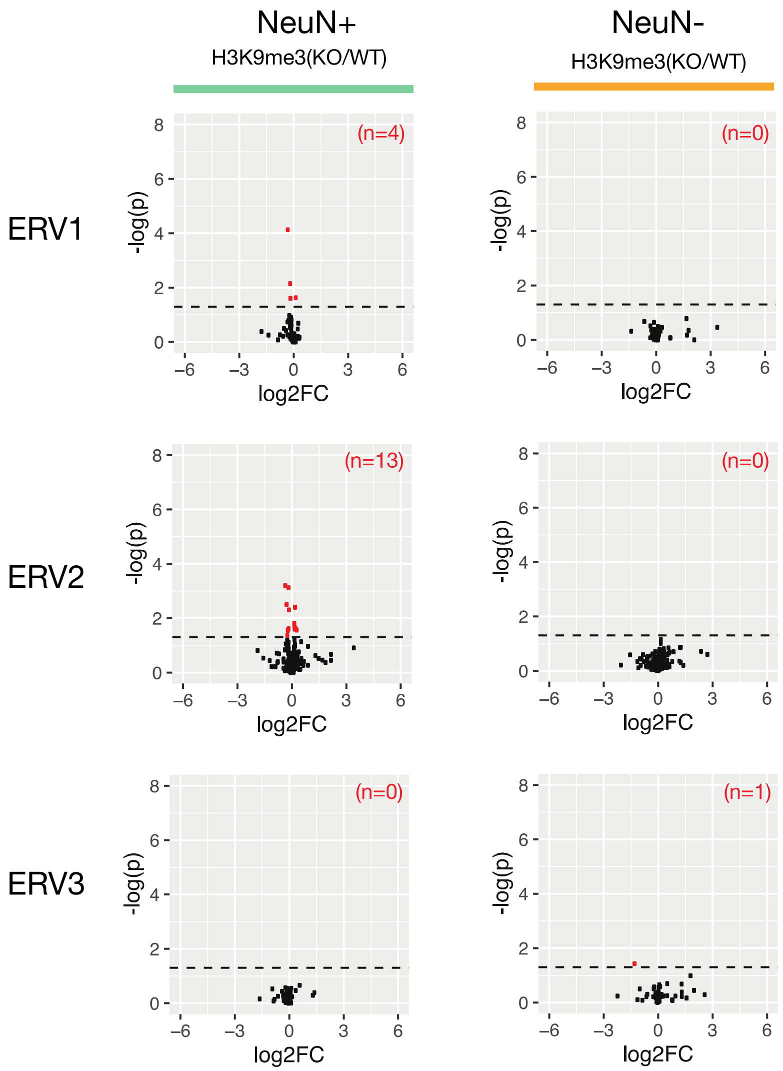

Figure S18. NeuN+ and NeuN- H3K9me3 (KO/WT) for ERV classes (I, II, and III). Scatterplot of H3K9me3 changes across NeuN+ in KO/WT and matched NeuN- for ERV1, ERV2, and ERV3. X-axis, log2FC H3K9me3 (KO/WT); y-axis -log(p-value) (n=3/group). Dashed lines indicate threshold for significance (two-sided, p-value = 0.05); all values higher than this line, highlighted in red, surpass this threshold. Total count of significant elements per ERV class noted in top right corner of each scatterplot.

Figure S19

Figure S19. NeuN+ and NeuN- H3K9me3 (KO/WT) for non-ERV repeat categories. Scatterplot of H3K9me3 changes across NeuN+ in KO/WT and matched NeuN- for repeat categories, as indicated. X-axis, log2FC H3K9me3 (KO/WT); y-axis -log(p-value) (n=3/group). Dashed lines indicate threshold for significance (two-sided, p-value = 0.05); all values higher than this line, highlighted in red, surpass this threshold. Total count of significant elements per repeat category noted in top right corner of each scatterplot.

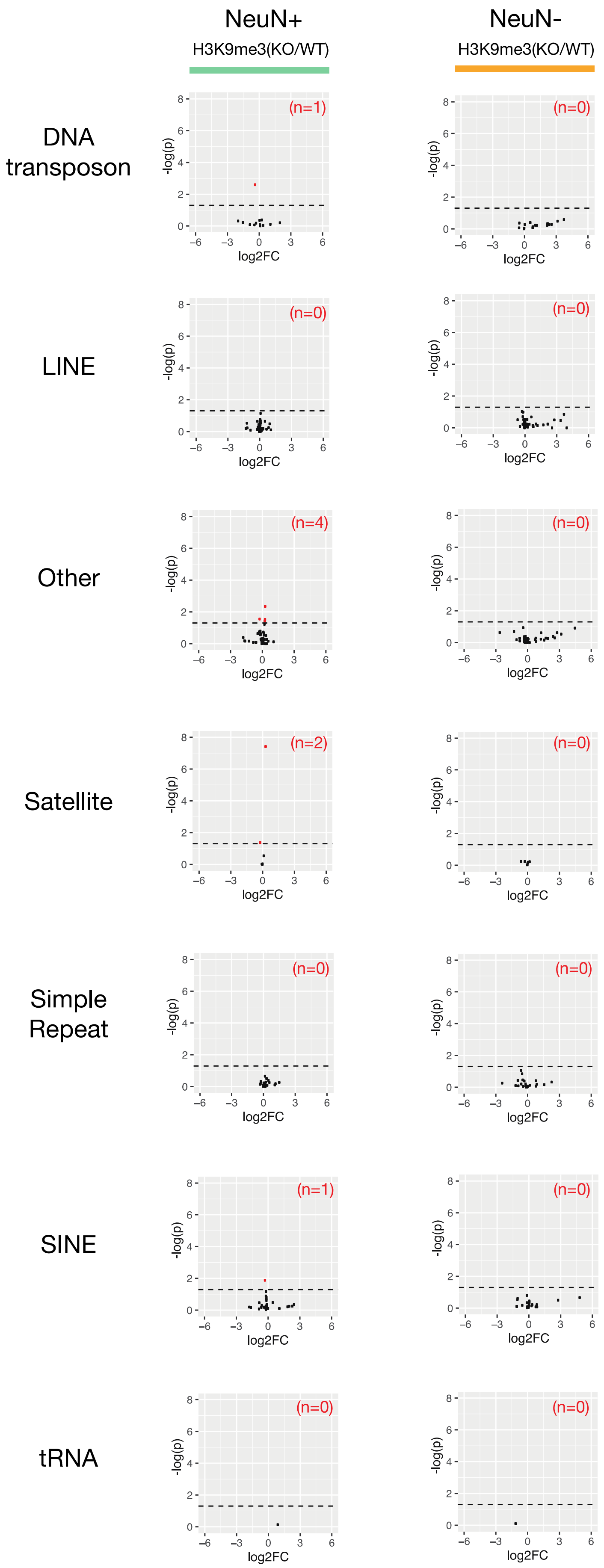

Figure S20

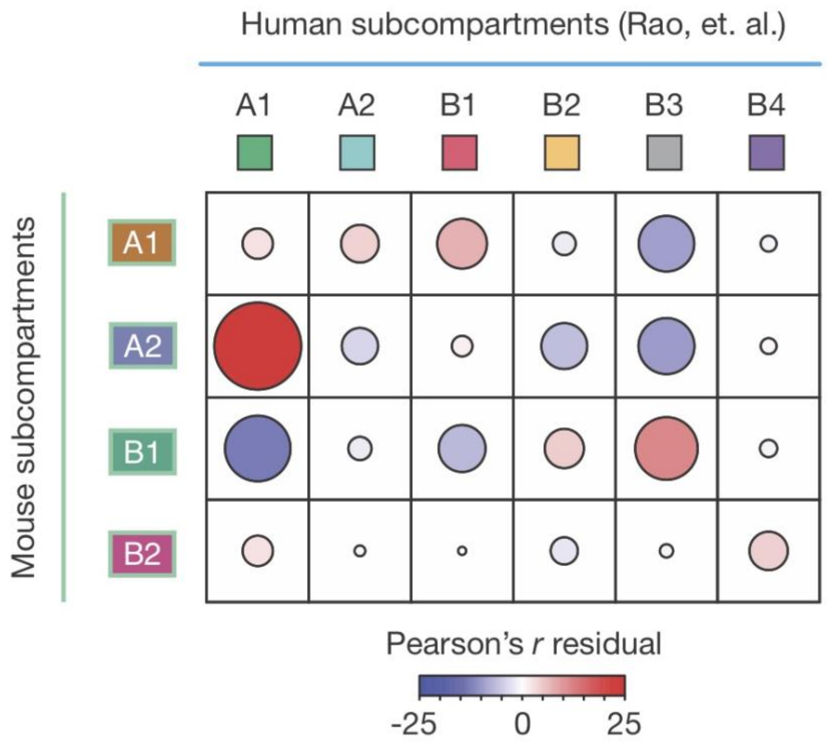

Figure S20. Mouse/human subcompartment comparisons. Observed/expected (Pearson's residual r) length of overlaps for lifted over coordinates from the human subcompartments (Rao, et. al. (2014))[11] with the NeuN+ subcompartments. The area of each ellipse represents $r_{absolute}$ ($|r|$), and color of each ellipse represents r, with a big blue circle representing substantially lower overlap than expected and a big red circle representing more overlap than expected based on length of the subcompartments under comparison.

Figure S21

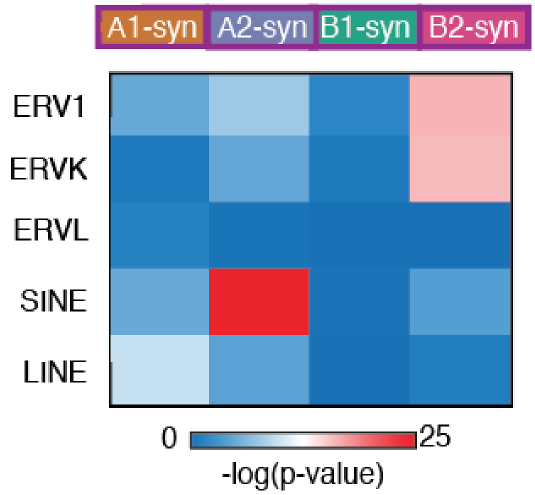

Figure S21. Inter-chromosomal contacts in human neurons are enriched with (H)ERV retroelements. Heatmap of significance of genome-wide associations (Fisher's 2x2 exact testing, one-sided) between syntenic genomic loci for each of the mouse subcompartments (denoted as "-syn") and repetitive element hotspots in humans (hg38). Notable associations include SINEs in A2-syn ($p=2.93 \times 10^{-42}$), and ERVK ($p=1.42 \times 10^{-16}$) and ERV1 ($p=6.01 \times 10^{-17}$) with B2-syn.

Supplementary Table S1. ChIP-Seq QC metrics

Histone PTM	Sex	Nuclei Type	Sequencing Depth	Total Reads
H3K9me3	Male	NeuN+	75bp, PE	80427273
	Male	NeuN-	75bp, PE	73018376
	Male	NeuN+	75bp, PE	85277944
	Male	NeuN-	75bp, PE	83575179
	Female	NeuN+	75bp, PE	85338301
	Female	NeuN-	75bp, PE	90128938
	Female	NeuN+	75bp, PE	83110870
	Female	NeuN-	75bp, PE	77285867
H3K27ac	Male	NeuN+	75bp, PE	93744420
	Male	NeuN-	75bp, PE	90871628
	Male	NeuN+	75bp, PE	94017026
	Male	NeuN-	75bp, PE	92256496
	Male	NeuN+	75bp, PE	100804952
	Male	NeuN-	75bp, PE	91303905
H3K79me2	Female	NeuN+	50bp, SE	28708757
	Female	NeuN-	50bp, SE	23407149
	Female	NeuN+	50bp, SE	18113433
	Female	NeuN-	50bp, SE	25284447

Supplementary Table S2. PacBio qPCR primers.

Name	Sequence
IAPEzi-f	5' CACGCTCCGGTAGAATACTTACAAAT 3'
IAPEzi-r	5' CCTGTCTAACTGCACCAAGGTAAAAT 3'
IAPEY3-f	5' ACAGAGGAGGACAACTGCTC 3'
IAPEY3-r	5' AACCTTACACAGGCAAAAGC 3'

Supplementary Table S3. PacBio oligonucleotide capture probes.

Sequence (5' to 3')	GC (%)
GAACTTTTCAGCTGGGGAACGAGAGTACCAGTGAGTACAGCTTTACGAGGTAAGTCTGGTCTTGAACTTTCTAAGGAAATTCAAGACAGTCTATCAGAAGTAAAGTGGAAAACGTGACCG	43.33
CAGCTCTTATATCCCACGAGGCTAGTCTCATTGCACGGGATAGAGTGAGTGTGCTTCAGCAGCCCGAGAGAGTTGCACGGCTAAGCACTGCAATGGAAAGGCTCTGCGGCATACGATGAG	54.17
ATTTGGAGATACACTTTGCTGTGGATTAGTGTTGCTTCTTTGATTGGTCTGTAAGCTTAAGGCCCAAACTAGGAGAGACAAGGTGGTTATTGCCCAGGCGCTTGCAGGACTAGAACATGG	45.83
TGTGTTATTTCCATTCAGTATGTTAAATTTACTAGGGCAGCTAATTTGTCAAAAAGTCTTTTTCAGTATATGTTACAGAATTGGACGGCTGAATTTGAACAGATCCTTCGGGAATTGAGA	34.17
CCCAATTGGATCTCCTCAGCATTTTCTTTCTTTAAAAAATGGGTGGGATTAATATTATTTGGAGATACACTTTGCTGTGGATTAGTGTTGCTTCTTTGATTGGTCTGTAAGCTTAAGGCC	37.5
AATTGAGACTTCAGGTCAACTCCACGCGCTTGGACCTGTCCCTGACCAAAGGATTACCCAATTGGATCTCCTCAGCATTTTCTTTCTTTAAAAAATGGGTGGGATTAATATTATTTGGAG	41.67
AGCCTGTGAGCCTAAGAGCTAATCCTGTACATGGCTCCTTTACCTACACACTGGGGATTTGACCTCTATCTCCACTCTCATTAATATGGGTGGCCTATTTGCTCTTATTAAAAGGAAAGG	44.17
TTAAGGCCCAAACTAGGAGAGACAAGGTGGTTATTGCCCAGGCGCTTGCAGGACTAGAACATGGAGCTTCCCCTGATAATATCTATGCTTAGGCAATAGGTCGCTGGCCACTCAGCTCTT	50
CAGCAGCCCGAGAGAGTTGCACGGCTAAGCACTGCAATGGAAAGGCTCTGCGGCATACGATGAGCCTATTCTAGGGAGACATGTCATCTTTCAGGAAGGTTCAGTGTCCTAGTTCCCTTC	52.5
TAGATGTATTATGACAAATAACTCAGCCGGGATGTGAACAAAAGTTTCCGGGATTGTGTGTTATTTCCATTCAGTATGTTAAATTTACTAGGGCAGCTAATTTGTCAAAAAGTCTTTTTC	34.17
GAACATGGAGCTTCCCCTGATAATATCTATGCTTAGGCAATAGGTCGCTGGCCACTCAGCTCTTATATCCCACGAGGCTAGTCTCATTGCACGGGATAGAGTGAGTGTGCTTCAGCAGCC	50.83
GATACAGAGAGGTCTCATGCTGGTTAATCAACTCATAGATCTTGTCCAGATACAACTAGATGTATTATGACAAATAACTCAGCCGGGATGTGAACAAAAGTTTCCGGGATTGTGTGTTAT	39.17
TCTTTTTCAGTATATGTTACAGAATTGGACGGCTGAATTTGAACAGATCCTTCGGGAATTGAGACTTCAGGTCAACTCCACGCGCTTGGACCTGTCCCTGACCAAAGGATTACCCAATTG	45
CGGTAATAAAGGTTCCCGTAAAGCAGACTGTTAAGAAGGATTCAACTGTATGAATTCAGAACTTTTCAGCTGGGGAACGAGAGTACCAGTGAGTACAGCTTTACGAGGTAAGTCTGGTCT	43.33
TGAACTCGGGACCTGGCGCAAGGAAGATCCCTCATTCCAGAACCAGAACTGCGGGTCGCGGTAATAAAGGTTCCCGTAAAGCAGACTGTTAAGAAGGATTCAACTGTATGAATTCAGAAC	48.33
ACTTTGTCAGCCTCAGTGACTACAGTCATAGATGAACAGGCCTCAGCTAATGTCAAGATACAGAGAGGTCTCATGCTGGTTAATCAACTCATAGATCTTGTCCAGATACAACTAGATGTA	41.67
TTCCCTTCCCACCAGGCAAAACGACACGGGAGCAGGTCAGGGTTGCTCTGGGTAAAAGCCTGTGAGCCTAAGAGCTAATCCTGTACATGGCTCCTTTACCTACACACTGGGGATTTGACC	53.33
ACGATGAGCCTATTCTAGGGAGACATGTCATCTTTCAGGAAGGTTCAGTGTCCTAGTTCCCTTCCCACCAGGCAAAACGACACGGGAGCAGGTCAGGGTTGCTCTGGGTAAAAGCCTGTG	52.5
TGATTCAGACAGCCTTGGCTCTGTCTGGACAGGTCCAGACGACTGACACCATTAACACTTTGTCAGCCTCAGTGACTACAGTCATAGATGAACAGGCCTCAGCTAATGTCAAGATACAGA	47.5

Supplementary Table S4. RNA-Seq metrics

Sample ID	Sex	Genotype	Sequencing Depth	Total Reads	Alignment (%)
2345	Male	WT	100bp, PE	70349554	93.37559979
2368	Male	WT	100bp, PE	75987918	92.62084533
2379	Male	WT	100bp, PE	63396242	92.7409325
2082	Female	WT	100bp, PE	62984976	91.8447472
2352	Female	WT	100bp, PE	55095462	93.69331725
2391	Female	WT	100bp, PE	60246206	93.9719059
2342	Male	KO	100bp, PE	71733102	93.39100936
2366	Male	KO	100bp, PE	72911448	92.97194866
2381	Male	KO	100bp, PE	76145870	92.17399184
2099	Female	KO	100bp, PE	62902030	91.9199269
2358	Female	KO	100bp, PE	65168926	91.45062787
2393	Female	KO	100bp, PE	62565724	92.2157953

Supplementary Table S5. Discordant + chimeric RNA-Seq

		Total alignments	Discordant (%)	Chimeric alignment
WT	1	70179376	0.004576	0
	2	77686089	0.003794	0
	3	60975124	0.003593	0
	4	84265199	0.004033	0
	5	69994089	0.003909	0
	6	67080807	0.005256	0
KO	1	71614047	0.004922	0
	2	81406241	0.003965	0
	3	74366605	0.003867	0
	4	82949120	0.004291	0
	5	85716155	0.004331	0
	6	71298531	0.005128	0
