# Supplementary material for: Neuron-specific chromosomal megadomain organization is adaptive to recent retrotransposon expansions
Source: Nat Commun. 2021 Dec 13;12:7243. doi: 10.1038/s41467-021-26862-z (PMC8669064; doi:10.1038/s41467-021-26862-z)
Supplement: Supplementary file 3 — Description of additional Supplementary File [file 41467_2021_26862_MOESM3_ESM.pdf]

Description of Additional Supplementary Files

File Name: Supplementary Data 1
Description: HiC QC metrics

File Name: Supplementary Data 2
Description: NeuN+ subcompartment genomic coordinates (GRCm38/mm10)

File Name: Supplementary Data 3
Description: NeuN- subcompartment genomic coordinates (GRCm38/mm10)

File Name: Supplementary Data 4
Description: Neuronal subcompartment genomic coordinates (GRCm38/mm10) [Espeso-Gil, et. al.]

File Name: Supplementary Data 5
Description: Neuronal subcompartment genomic coordinates (GRCm38/mm10) [Fernandez-Albert, et. al.]

File Name: Supplementary Data 6
Description: Neuronal subcompartment genomic coordinates (GRCm38/mm10) [Bonev, et. al.]

File Name: Supplementary Data 7
Description: ESC subcompartment genomic coordinates (GRCm38/mm10) [Bonev, et. al.]

File Name: Supplementary Data 8
Description: NeuN+ trans interactions

File Name: Supplementary Data 9
Description: NeuN- trans interactions

File Name: Supplementary Data 10
Description: Repeat Sequence Classification

File Name: Supplementary Data 11
Description: PacBio SMRT long-read sequencing QC metrics

File Name: Supplementary Data 12
Description: PacBio full-length coordinates (de novo)

File Name: Supplementary Data 13
Description: Differential trans interactions Setdb1-deficient and control neurons with wildtype Setdb1 levels (n=4/group, 1Mb resolution, padj < 0.05)

File Name: Supplementary Data 14
Description: Differential cis interactions Setdb1-deficient and control neurons with wildtype Setdb1 levels (1Mb resolution, padj < 0.05)

File Name: Supplementary Data 15
Description: Differentially expressed RNAs in Setdb1 mutant vs. control cortex (n=6/group, two-sided, padj < 0.05)

File Name: Supplementary Data 16
Description: Differential DNA repeats of RNA-Seq from Setdb1-deficient and control microglia (two-sided, p < 0.05)

File Name: Supplementary Data 17
Description: Differential genes list of RNA-Seq from Setdb1-deficient and control microglia (n=3/group, one-sided, p < 0.05)

File Name: Supplementary Data 18
Description: Differential peaks list of ATAC-seq from Setdb1-deficient and control microglia (n=3/group, two-sided, p < 0.05)

File Name: Supplementary Data 19
Description: Differential H3K9me3 enrichment across repetitive elements in NeuN+ from Setdb1-deficient and littermate controls (n=3/group, two-sided, p < 0.05)

File Name: Supplementary Data 20
Description: Differential H3K9me3 enrichment across repetitive elements in NeuN- from Setdb1-deficient and littermate controls (n=3/group, two-sided, p<0.05)
