# Supplementary material for: Neuron-specific chromosomal megadomain organization is adaptive to recent retrotransposon expansions
Source: Nat Commun. 2021 Dec 13;12:7243. doi: 10.1038/s41467-021-26862-z (PMC8669064; doi:10.1038/s41467-021-26862-z)
Supplement: Supplementary file 24 — Reporting Summary [file 41467_2021_26862_MOESM24_ESM.pdf]

nature research

Corresponding author(s): Schahram Akbarian

Last updated by author(s): Aug 10, 2021

Reporting Summary

Nature Research wishes to improve the reproducibility of the work that we publish. This form provides structure for consistency and transparency in reporting. For further information on Nature Research policies, see our Editorial Policies and the Editorial Policy Checklist.

Statistics

For all statistical analyses, confirm that the following items are present in the figure legend, table legend, main text, or Methods section.

n/a	Confirmed	
☐	☒	The exact sample size (*n*) for each experimental group/condition, given as a discrete number and unit of measurement
☐	☒	A statement on whether measurements were taken from distinct samples or whether the same sample was measured repeatedly
☐	☒	The statistical test(s) used AND whether they are one- or two-sided<br>*Only common tests should be described solely by name; describe more complex techniques in the Methods section.*
☐	☒	A description of all covariates tested
☐	☒	A description of any assumptions or corrections, such as tests of normality and adjustment for multiple comparisons
☐	☒	A full description of the statistical parameters including central tendency (e.g. means) or other basic estimates (e.g. regression coefficient) AND variation (e.g. standard deviation) or associated estimates of uncertainty (e.g. confidence intervals)
☐	☒	For null hypothesis testing, the test statistic (e.g. *F*, *t*, *r*) with confidence intervals, effect sizes, degrees of freedom and *P* value noted<br>*Give P values as exact values whenever suitable.*
☐	☒	For Bayesian analysis, information on the choice of priors and Markov chain Monte Carlo settings
☐	☒	For hierarchical and complex designs, identification of the appropriate level for tests and full reporting of outcomes
☐	☒	Estimates of effect sizes (e.g. Cohen's *d*, Pearson's *r*), indicating how they were calculated

Our web collection on statistics for biologists contains articles on many of the points above.

Software and code

Policy information about availability of computer code

Data collection	Figures 1, 2, 3, 4: No software was used for data collection.<br><br>Figure 4: Images were acquired using the Olympus VS120 fluorescence microscope.<br><br>Figure 5: RNA FISH and immunohistochemistry images were acquired using a Zeiss LSM780 confocal microscope. Electron microscopy images were acquired using the Hitachi 7000 electron microscope.
Data analysis	Figure 1: Samples were sorted using the BD FACSDiva (v6.1) software. Hi-C datasets were analyzed using HiC-Pro(v2.9) after alignment with bowtie2.2, Juicer, and HOMER(v4.8) after alignment with bwa-mem(v0.7.15). Subcompartment calls were performed using packages available on R 3.6.0 and RStudio (v1.1.463). Statistical testing for genome-wide associations were performed using the fisher.exact() function on R. ChIP-Seq tracks were aligned using bowtie2.2, processed with samtools/0.1.19, and visualized using IGV(v2.3.90).<br><br>Figure 2: Hi-C datasets were processed using the tools indicated above.<br><br>Figure 3: PacBio long-read sequencing was analyzed using packages included with SMRT Link(v5.1.0) and blast+(v.2.7.1).<br><br>Figure 4: Transcriptomics were processed using Tophat2.1.1 and HTSeq (v0.11.2), as well as the voomlimma R package. GO enrichments were determined using the ClueGO(v2.5.1) application through Cytoscape(v3.6.1).<br><br>Figure 5: IHC images were analyzed using ImageJ (v1.49u) (NIH) and statistically interpreted using Prism 7. Transcriptomics were processed using Tophat2.1.1, samtools(v1.9), and featureCounts (v1.6.3). GO enrichments were determined using ShinyGO(v0.61). ATAC-Seq was analyzed using MACS2(2.2.7.1) (peak calling) and diffBind (v3.13) (differential analysis).

Figure 6: No software was used in data analysis; IAPs were visually quantified by a blinded observer during data collection for subsequent statistical interpretation.

Scripts used for bioinformatics analyses will be are available at https://github.com/sandchand5/hic-neuron-erv (doi: 10.5281/zenodo.5549985).

For manuscripts utilizing custom algorithms or software that are central to the research but not yet described in published literature, software must be made available to editors and reviewers. We strongly encourage code deposition in a community repository (e.g. GitHub). See the Nature Research guidelines for submitting code & software for further information.

Data

Policy information about availability of data

All manuscripts must include a data availability statement. This statement should provide the following information, where applicable:

- Accession codes, unique identifiers, or web links for publicly available datasets
- A list of figures that have associated raw data
- A description of any restrictions on data availability

Sequencing data for genome-scale analysis (Hi-C, ChiP-Seq, RNA-Seq, PacBio SMRT-Seq) have been deposited in NCBI's Gene Expression Omnibus (GSE168524) and will be made accessible prior to publication. Other publicly available datasets used in this paper include the following: Mouse Encode Project ((http://chromosome.sdsc.edu/mouse/download.html), H3K27ac (GSE99363), H3K79me2 (https://www.ncbi.nlm.nih.gov/sra/SRP154319), Hi-C datasets (Espeso-Gil, et. al. (https://www.ncbi.nlm.nih.gov/sra/SRP154319), Fernandez-Albert, et. al. (GSE125068), and Bonev, et. al. (GSE96107)).

Field-specific reporting

Please select the one below that is the best fit for your research. If you are not sure, read the appropriate sections before making your selection.

☒ Life sciences ☐ Behavioural & social sciences ☐ Ecological, evolutionary & environmental sciences

For a reference copy of the document with all sections, see nature.com/documents/nr-reporting-summary-flat.pdf

Life sciences study design

All studies must disclose on these points even when the disclosure is negative.

Sample size

The number of animals needed was calculated based on the absolute minimum number of nuclei required to complete each experiment while balancing for sex, accounting for experimental costs, and minimizing technical variation.

Figure 1: Hi-C (n=4 — 2F/2M); ChIP-Seq (H3K9me3 (n=4 — 2F/2M), H3K27ac* (n=3 — 3M), H3K79me2* (n=2 — 2F). *These datasets were used from previously published papers from our lab (PMID 28671686, https://doi.org/10.1101/2021.01.31.428988).

Figure 2: Hi-C (n=2 — 1F/1M [SPRET/EiJ], n=2 — 1F/1M [C57/BL6J])

Figure 3: PacBio (n=7 — 5M/2F).

Figure 4: Hi-C (n=4 — 2F/2M [KO]); RNA-Seq (n=6 — 3F/3M).

Figure 5: IHC (n=6 — 3F/3M [WT], n=6 — 3F/3M [KO]); RNA-Seq (n=3 — 2F/1M [WT], n=3 — 2F/1M [KO]); ATAC-Seq (n=3 — 2F/1M [WT], n=3 — 2F/1M [KO])

Figure 6: WB (n=2 — 1F/1M [WT], n=2 — 1F/1M [KO]); EM (n=4 — 2F/2M [WT], n=4 — 2F/2M [KO])

Data exclusions

No data was excluded from any of the studies included in this submission.

Replication

Experimental findings for next-generation sequencing data obtained from group analyses were additionally verified by observing individual sample files in each group to ascertain reproducibility of the conclusions across each replicate. All such attempts were deemed successful prior to inclusion in this submission.

In addition to these validation measures, HI-C experiments were performed in two batches (NeuN+/NeuN-) and two batches (SPRET/Eij/C57), and reproducible. ChIP-Seq H3K9me3 findings were reproducible across three different experiments, and RNA-Seq findings were reproducible across 3 different experiments. PacBio samples were processed in batches across three different experiments, with reproducible results. IHC experiments were reproducible across three independent experiments, Western blotting was reproducible across 3-5 experiments (Figure 6d, 5d respectively). EM experiments were reproducible across two batches.

Randomization

Randomization was not relevant to our study as animal cohorts of specific genotypic/strain backgrounds were selected for predetermined purposes as described above.

Blinding

All next-generation sequencing data, although unblinded, was processed using unbiased algorithms and scripts via the Unix and R platforms. Quantifications of IAPs in the electron microscopy images were done by a blinded experimenter during imge acquisition. Blinding was not possible during data analysis (i.e., statistical testing) as group designation had to be known in order to appropriately assess significance across

groups. For all other experiments, investigators were blinded during wet lab prep with alphanumerical designations used for all samples. Similarly, investigators were blinded during bioinformatics processing as all samples were directly fed into a pre-written algorithmic pipeline. All IHC images were also acquired by a blinded observer.

Reporting for specific materials, systems and methods

We require information from authors about some types of materials, experimental systems and methods used in many studies. Here, indicate whether each material, system or method listed is relevant to your study. If you are not sure if a list item applies to your research, read the appropriate section before selecting a response.

Materials & experimental systems

n/a	Involved in the study	
☐	☒	Antibodies
☒	☐	Eukaryotic cell lines
☒	☐	Palaeontology and archaeology
☐	☒	Animals and other organisms
☒	☐	Human research participants
☒	☐	Clinical data
☒	☐	Dual use research of concern

Methods

n/a	Involved in the study	
☐	☒	ChIP-seq
☐	☒	Flow cytometry
☒	☐	MRI-based neuroimaging

Antibodies

Antibodies used

Figure 1: anti-NeuN antibody (clone A60, Alexa Fluor 488 conjugated; EMD Millipore Corp., MAB377X (1:1000)); anti-H3K9me3 (Abcam, ab8898); anti-H3K27ac (Active Motif, #39133); anti-H3K79me2 (Abcam, ab3594).

Figure 5: anti-Iba1 (Abcam, ab178846, 1:1500); anti-GFAP (Abcam, ab4674, 1:1000); anti-actin (Sigma, A2066, 1:100); anti-NeuN-Alexa555 (Millipore, MAB377X) (1:200); Tomato-lectin (Vector, DL-1174, 1:100); CD11b-FITC Monoclonal Antibody (M1/70) (eBioscience, 11-0112-81) (1:2000).

Figure 6: anti-IAP-gag (a gift from Dr. Bryan R. Cullen, Duke University, developed in-house) (1:10000); goat anti-rabbit HRP (Invitrogen, #31460) (1:5000).

Validation

The antibodies referenced in Figure 1 (anti-NeuN, anti-H3K9me3, anti-H3K27ac, anti-H3K79me2) and Figure 5 (anti-Iba1, anti-GFAP, anti-actin, anti-NeuN-Alexa555, Tomato-lectin, CD11b-FIC Monoclonal Antibody)have each been validated in several publications as noted on the respective manufacturer's websites. The anti-IAP gag antibody referenced in Figure 6 has been used and validated in several publications (PMID 22991445, 25457166, 26546670).

Animals and other organisms

Policy information about studies involving animals; ARRIVE guidelines recommended for reporting animal research

Laboratory animals

Figure 1: C57/BL6J mice (M&F, 3-9mos)

Figure 2: SPRET/EiJ mice (M&F, 3-9mos); C57/BL6J mice (M&F, 3-9mos)

Figure 3: C57/BL6J mice (M&F, 3-9mos), 129S1/C57 mice (M&F, 3-9mos)

Figures 4, 5, 6: WT & KO mice are C57/BL6J mice (M&F, 3-9mos); KO mice are CamK-Cre+,Setdb12lox/2lox mouse strain.

Wild animals

The study did not involve wild animals.

Field-collected samples

The study did not involve samples collected from the field.

Ethics oversight

All mouse work detailed in this submission was approved by the Institutional Animal Care and Use Committee (IACUC) of the Icahn School of Medicine at Mount Sinai. Protocol LA12-00222.

Note that full information on the approval of the study protocol must also be provided in the manuscript.

ChIP-seq

Data deposition

☒ Confirm that both raw and final processed data have been deposited in a public database such as GEO.

☒ Confirm that you have deposited or provided access to graph files (e.g. BED files) for the called peaks.

Data access links
May remain private before publication.

GEO for H3K27ac raw/processed data (GSE99363)
Other data (see below) (GSE168524)

Files in database submission

H3K9me3 ChIP-Seq NeuN+ (pos) and NeuN- (neg):
FEM1-pos.R1.uniqmapped.rmdup.bam:
FEM1-neg.R1.uniqmapped.rmdup.bam
FEM2-pos.R1.uniqmapped.rmdup.bam
FEM2-neg.R1.uniqmapped.rmdup.bam
WT2-pos.R1.uniqmapped.rmdup.bam
WT2-neg.R1.uniqmapped.rmdup.bam
WT3-pos.R1.uniqmapped.rmdup.bam
WT3-neg.R1.uniqmapped.rmdup.bam

diffReps files:
OV+-_1kb: H3K9me3
OVNG_1kb: H3K27ac
diffReps_Neurons_v_glia_1kb: H3K79me2

Genome browser session
(e.g. UCSC)

See above.

Methodology

Replicates

H3K9me3 (n=4)
H3K27ac* (n=3)
H3K79me2* (n=2)

*These datasets were used from previously published papers from our lab (PMID 28671686, https://doi.org/10.1101/2021.01.31.428988).

Sequencing depth

H3K9me3: HiSeq 2500, 75bp, paired-end
H3K27ac: HiSeq 2000, 75bp, paired-end
H3K79me2: HiSeq 2000, 75bp, paired-end

Antibodies

anti-H3K9me3 (Abcam, ab8898)
anti-H3K27ac (Active Motif, #39133)
anti-H3K79me2 (Abcam, ab3594)

Peak calling parameters

No peaks were called during these ChIP-Seq analyses. Instead, regions of significant difference between denoted treatment and control conditions (NeuN+ and NeuN-, respectively) samples were called using the program diffReps (1kb resolution, $p<0.001$).

Data quality

diffReps implements a sliding scale binning strategy to identify regions of significant difference between treatment and control groups and does not identify discrete peaks. Instead, contiguous regions of the genome, 1kb or greater in length, with significant differences between the two groups are reported as discrete entries.

Treatment: NeuN+; Control: NeuN-

H3K9me3: 36137
H3K27ac: 146538
H3K79me2: 101147

Software

diffReps (PMID 23762400) was used for these analyses using default parameters (1 kb resolution, $p<0.001$).

Flow Cytometry

Plots

Confirm that:

☒ The axis labels state the marker and fluorochrome used (e.g. CD4-FITC).

☒ The axis scales are clearly visible. Include numbers along axes only for bottom left plot of group (a 'group' is an analysis of identical markers).

☒ All plots are contour plots with outliers or pseudocolor plots.

☒ A numerical value for number of cells or percentage (with statistics) is provided.

Methodology

Sample preparation

Cortical tissue was prepped for fluorescence activated nuclear sorting (FANS) as follows. Briefly, the dissected tissue was homogenized in a hypotonic lysis solution and fixed in 1% formaldehyde for 10 minutes at room temperature. The cross-

	linking reaction was quenched with 125 mM glycine. The nuclei were then purified by centrifugation at 4000xg and resuspended in a 1:1 solution of the hypotonic lysis solution and a 1.8M sucrose solution prior to re-centrifugation at 4000xg to isolate out cortical nuclei. The pellet was then resuspended in Dulbecco's phosphate buffered saline (DPBS) containing 0.1% BSA and 1:1000 anti-NeuN antibody (clone A60, Alexa Fluor 488 conjugated; EMD Millipore Corp., MAB377X). Samples were incubated for 45 minutes while rotation and protected from light at 4C. DAPI (Invitrogen) was added immediately before sorting to label all nuclei.
Instrument	Sorting was performed at the Flow Cytometry CoRE at the Icahn School of Medicine at Mount Sinai using the BD FACSAria III cell sorter.
Software	The BD FACSDiva (v6.1) software was used to collect and analyze the flow cytometry data.
Cell population abundance	NeuN+ and NeuN- population gating was determined empirically by visual inspection for each sorting experiment, resulting in an approximate ratio of 3:1 NeuN+ : NeuN- nuclei from mouse cortical tissue.
Gating strategy	Nuclei were collected as NeuN+ and NeuN- populations following serial gating (FSC, SSC, DAPI, NeuN) and pelleted for downstream experimental processing.

☒ Tick this box to confirm that a figure exemplifying the gating strategy is provided in the Supplementary Information.
